# Cellular Localization of the Herpes Simplex Virus ICP0 Protein Dictates Its Ability to Block IRF3-Mediated Innate Immune Responses

**DOI:** 10.1371/journal.pone.0010428

**Published:** 2010-04-29

**Authors:** Patrick Paladino, Susan E. Collins, Karen L. Mossman

**Affiliations:** Department of Pathology and Molecular Medicine, Institute for Infectious Disease Research, McMaster University, Hamilton, Ontario, Canada; The University of Chicago, United States of America

## Abstract

Interferon regulatory factor 3 (IRF3) is important for innate antiviral responses; accordingly, many viruses target and inactivate IRF3. The ability of the Herpes simplex virus type 1 (HSV-1) immediate early protein ICP0 to inhibit IRF3 is controversial and has not been studied solely in the context of a wild type HSV-1 infection. Discrepancies in the literature surround the mechanism by which ICP0 antagonizes the IRF3 pathway, the cellular localization of ICP0 inhibitory activity and the ability of ICP0 to interfere with interferon and interferon-stimulated gene induction. In this study, we set out to investigate the role of ICP0 localization and the requirement of the proteasome during the inhibition of IRF3 activation within the context of an HSV-1 infection. Collectively, the results presented herein demonstrate that incoming wild type HSV-1 activates IRF3 and that *de novo* produced ICP0 prevents sustained IRF3 activation following its translocation from the nucleus to the cytoplasm. While previous studies implicate the E3 ubiquitin ligase domain of ICP0 in mediating its biological functions, including the inhibition of IRF3, we show that cytoplasmic ICP0 does not require the proteasome for this activity. Instead, proteasome function is required to localize ICP0 to the cytoplasm where it mediates its inhibitory effect independent of E3 ubiquitin ligase activity. The importance of these findings is discussed within the context of an HSV-1 infection.

## Introduction

Interferon (IFN) regulatory factor 3 (IRF3) is a constitutively expressed transcription factor that controls the expression of IFN and IFN-stimulated genes (ISGs) following virus recognition. All identified innate immune receptors are capable of signaling through IRF3 to mediate their antiviral effects, including toll-like receptors (TLRs), retinoic acid-inducible gene I (RIG-I)-like receptors and DNA sensors such as DAI [1], [2]. In addition, the entry of enveloped virus particles, such as HSV-1, elicits an IRF3-dependent but IFN-independent cellular antiviral response [3], [4], [5], [6]. As a result, there are several avenues the host defense machinery can employ to activate IRF3-mediated antiviral responses. The activation of IRF3 has been intensively studied and is reviewed in detail elsewhere [7], [8], [9]. The hallmarks of IRF3 activation are phosphorylation by TANK-binding kinase-1 (TBK-1) or I kappa B kinase ε (IKKε), dimerization and nuclear translocation. Following its activation, IRF3 is degraded via the ubiquitin-proteasome pathway [10], [11]. The critical nature of IRF3 was shown in studies where its absence results in abrogation of innate antiviral responses [4], [12], [13]. Accordingly, diverse viruses encode proteins that inhibit IRF3 activation [14].

HSV-1 is an enveloped, dsDNA virus that is capable of undergoing both lytic and latent infections within its host. The success of HSV-1 as a human pathogen can, in part, be attributed to the temporal expression of proteins that combat host antiviral barriers. ICP0 is a multifunctional, immediate-early protein that plays a pivotal role during lytic and latent infections [15], [16], [17] due in part to evasion of host IFN responses [18]. Upon infection with HSV-1, ICP0 localizes to the nucleus (1–4 hours), then the cytoplasm (6–8 hours) and then shuttles between the two compartments [19], [20], [21], [22]. ICP0 interacts with several cellular and viral proteins, ultimately mediating its effects by regulating gene expression, cell cycle progression and protein expression [16], [17].

ICP0 possesses several functional domains including a RING finger domain which mediates E3 ubiquitin ligase activity [23], [24], [25] and is thought to be required for all but one of ICP0's known functions, namely its ability to modulate rRNA degradation [26]. Experimentally, the E3 ubiquitin ligase activity of ICP0 is assessed through the use of RING finger mutants such as FXE [27] or by employing proteasome inhibitors such as MG132. As ICP0 is thought to function primarily within the nucleus, the role of localization in dictating the function of ICP0 has been understudied. However, ICP0 undergoes various posttranslational modifications within different subcellular compartments [28], [29], [30], [31], suggesting that ICP0 may possess different activities within different cellular locations.

With respect to evasion of host innate responses, ICP0 was first shown to interfere with IFN-mediated inhibition of HSV-1 transcription [5], [32]. Subsequent studies found that amongst all immediate-early genes, ICP0 is responsible for dampening the production of IFN and ISGs during infection [33], [34]. Although experimentally the RING finger of ICP0 was required for this activity, degradation of known IRF3 components was not observed. Studies employing a co-infection model with HSV-1 and Sendai virus (SeV) found that ICP0 inhibited IRF3 nuclear translocation and mediated its degradation [35], [36]. Within the same model, nuclear ICP0 sequestered IRF3 and CBP/p300 away from host chromatin, preventing ISG and IFN induction [35]. In an apparent contradiction, expression of ICP0 from a tetracycline-inducible plasmid system, which restricts ICP0 expression to the nucleus, failed to block ISG induction following IFN or dsRNA treatment [37].

Given the apparent contradictory results regarding the role of ICP0 in combating IRF3-mediated responses, we investigated the mechanism by which ICP0 blocks IRF3 activity within the context of an HSV-1 infection. We employed a non-immortalized, non-transformed primary fibroblast cell line that is fully competent for IRF3 signaling and is susceptible and permissive to infection by HSV-1. This approach avoids the complications that result from using reporter assays or co-infection models and takes into account the recent appreciation that diverse viruses and viral ligands differentially modify and activate IRF3 [3], [38], [39] and that interferon pathways are altered following cellular immortalization [40]. We carefully monitored the localization of ICP0 as recent studies have begun to address the biological relevance of its localization during the course of an HSV-1 infection [19], [35], [37], [41], [42]. IRF3 activation was monitored by native western blots to examine IRF3 dimerization, phospho-specific antibodies to assess IRF3 phosphorylation and microscopy to monitor IRF3 localization. ISG induction was examined to corroborate the activation status of IRF3 following HSV-1 infection. Collectively, the data presented herein demonstrate that IRF3 is activated upon infection with WT HSV-1. However, during the course of infection ICP0 inhibits IRF3 phosphorylation, dimerization and nuclear translocation when localized in the cytoplasm but has no apparent inhibitory activity when located within the nucleus. Surprisingly, our data also suggest that the E3 ubiquitin ligase activity associated with the RING finger domain is not directly required to inhibit the activation of IRF3 but instead plays a critical role in modulating the cellular localization of ICP0.

## Results

### ICP0 inhibits IRF3 activation and ISG induction at later stages of infection

Previously, characterization of the cellular response to enveloped virus particle entry utilized cycloheximide, UV-inactivation or genetic inactivation to study IRF3 function in the absence of virus replication and/or *de novo* protein production. To assess IRF3 activation during the course of a WT HSV-1 infection and examine the effects of ICP0, we conducted a time course experiment examining IRF3 dimerization and nuclear translocation following infection with WT HSV-1 and a corresponding ICP0-null mutant in non-immortalized, non-transformed human fibroblasts. To correlate IRF3 activation with a functional antiviral response, induction of ISG56 was examined by western blot analysis, as this protein is induced rapidly and efficiently during an IRF3-dependent antiviral response [12], [43]. Infection with SeV served as a positive control for IRF3 activation and ISG56 induction [43], [44].

**Figure 1:**
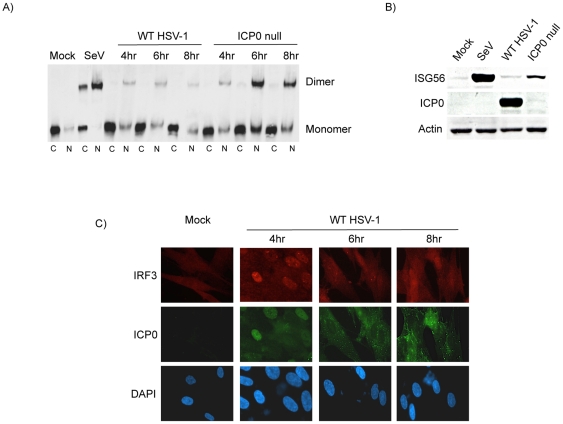
ICP0 inhibits the sustained activation of IRF3 during the later stages of an HSV-1 infection. (A) HEL cells were mock treated, infected with WT HSV-1 (strain F) or a corresponding ICP0 null virus (R7910). Cytoplasmic and nuclear protein extracts were resolved using native western blotting to assess IRF3 dimerization. (B) Western blot analysis of whole cell protein lysates collected from HEL cells after 8 hours of infection as indicated. (C) Immunofluorescence microscopy examining ICP0 and IRF3 subcellular localization following a time course infection of HEL cells with WT HSV-1. Cell nuclei were hoechst stained (DAPI). A representative field of view is shown for mock treated cells. In all cases, SeV served as a positive control for activation of the IRF3 pathway.

IRF3 dimers were absent in mock-infected samples but present in both the cytoplasmic and nuclear fractions following SeV infection (Figure 1A). Upon infection with WT HSV-1 (strain F), nuclear IRF3 dimers were observed at 4 and 6 hours post-infection (hpi) but diminished at later times. Following infection with a corresponding ICP0-null HSV-1 (R7910), IRF3 dimerization and nuclear translocation were sustained up to 8 hours. In agreement with the activation of IRF3, ISG56 induction was detected following infection with SeV or ICP0-null HSV-1 but not with WT HSV-1 (Figure 1B). Similar results were observed with additional strains of HSV-1, namely KOS and 17syn, and their ICP0 null counterparts, n212 and dl1403, respectively (data not shown). Similar to previous studies [19], [21], [22], ICP0 was found to initially localize to the nucleus and subsequently translocate to the cytoplasm. Immunofluorescence microscopy performed in parallel with the western blots confirmed the cellular localization of ICP0 and the activation status of IRF3 following WT HSV-1 infection (Figure 1C). In summary, these results demonstrate that upon infection with WT HSV-1, IRF3 is activated but is subsequently inhibited by ICP0 at a time point that correlates with its cytoplasmic localization. Furthermore, the transient induction of IRF3 following infection with WT HSV-1 is insufficient to promote accumulation of ISG56 protein.

### Cytoplasmic, but not nuclear, ICP0 mediates the inhibition of IRF3

To date, the biological activities ascribed to ICP0 occur early in infection when ICP0 is localized to the nucleus. Despite evidence that ICP0 blocks IRF3 activity [34], [35], [36], nuclear restricted ICP0 produced by plasmid transfection fails to dampen IRF3-mediated IFN production [37]. Given our finding that inhibition of IRF3 activation during a WT HSV-1 infection correlates with ICP0 cytoplasmic localization, we employed different experimental systems to limit the subcellular localization of ICP0 during HSV-1 infection to address the hypothesis that cytoplasmic ICP0 inhibits IRF3 activation. In this regard, IRF3 activation was examined following infection with HSV-1 mutant viruses that restrict ICP0 expression to either the nucleus (R7914; F strain) or the cytoplasm (D8; 17syn strain) or following HSV-1 infection in the presence of chemical inhibitors that restrict the expression of ICP0 within the nuclear compartment. Lastly, since previous studies examined the ability of ICP0 to inhibit IRF3 following activation by SeV [35], [36] or polyI:C [37], we investigated whether ICP0 localization plays a role in its ability to inhibit IRF3 activated by different stimuli.

**Figure 2:**
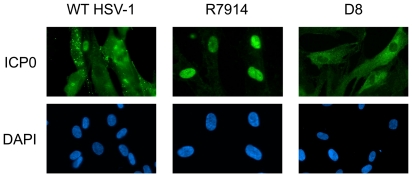
ICP0 expressed from the D8 mutant localizes exclusively in the cytoplasm. HEL cells were infected with WT HSV-1 (F strain), R7914 or D8 for 8 hours. ICP0 localization was monitored by immunofluorescence microscopy. Cell nuclei were hoechst stained (DAPI).

**Figure 3:**
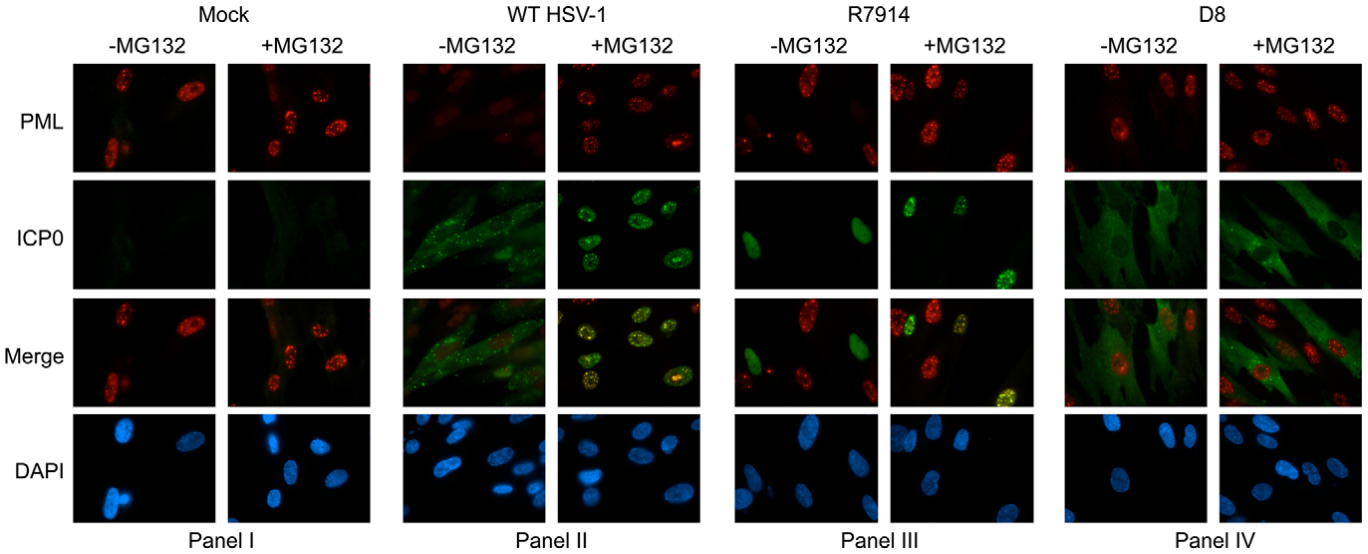
Inhibition of the proteasome precludes PML degradation during WT or ICP0 mutant HSV-1 infection. HEL cells were treated with WT HSV-1 or ICP0 mutant viruses, R7914 or D8, for 8 hours in the presence or absence of MG132. ICP0 and PML expression was examined using immunofluorescence microscopy. Cell nuclei were labelled with a hoechst stain (DAPI). A representative field of view is shown for mock treated cells.

**Figure 4:**
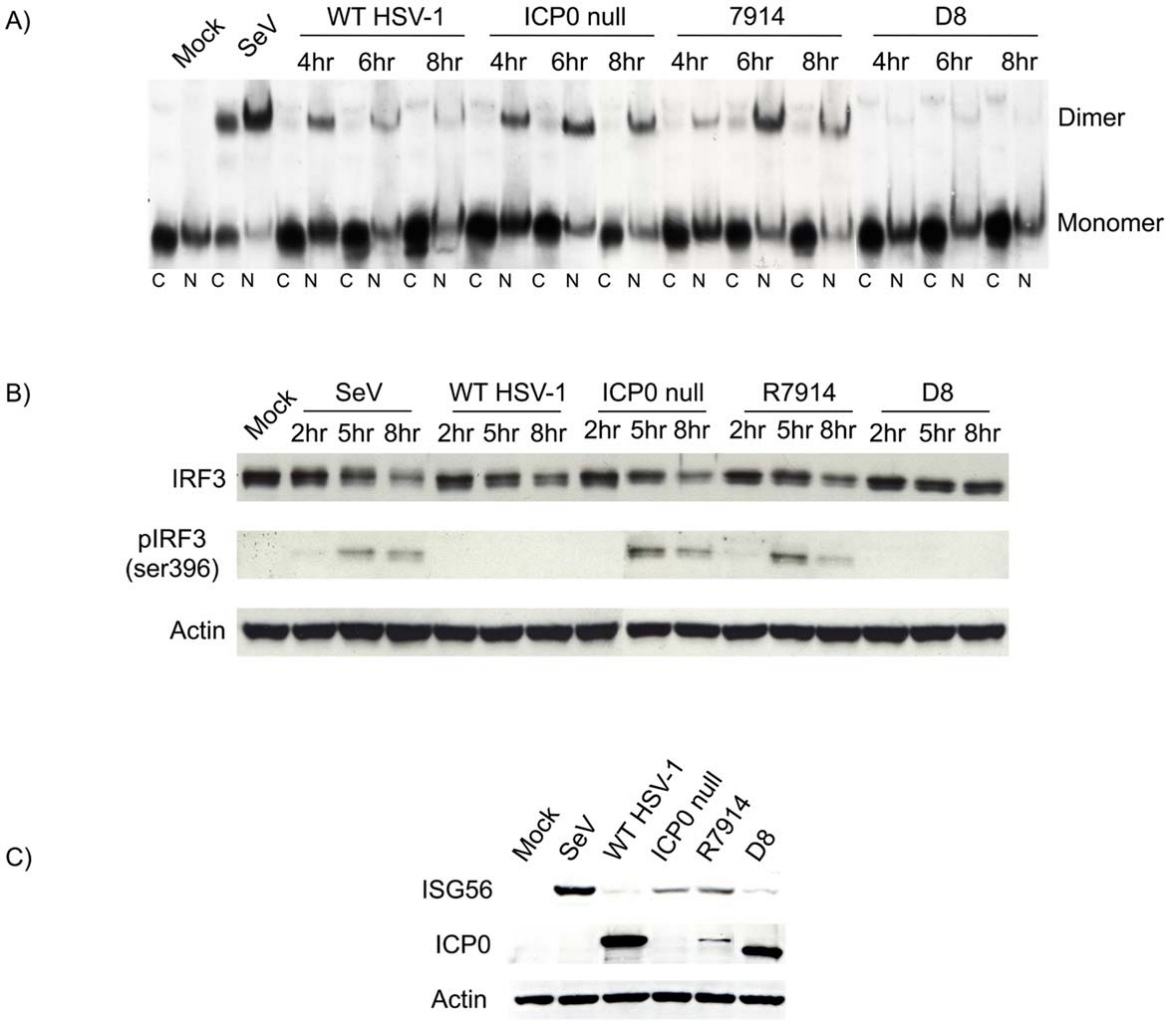
Cytoplasmic but not nuclear ICP0 inhibits IRF3 activation and subsequent ISG induction. (A) HEL cells were infected with WT HSV-1 (strain F), ICP0 null HSV-1 (R7910), R7914, or D8 for the indicated times. Cytoplasmic and nuclear protein extracts were resolved using native western blots to examine IRF3 dimerization. (B) HEL cells were infected as denoted in part A. Total and phospho-IRF3 (ser-396) levels were examined by western blotting. (C) ISG56 and ICP0 expression were measured using western blot analysis following an 8 hour infection as indicated. In all cases, SeV served as a positive control for activation of the IRF3 pathway.

Infection of fibroblasts with R7914 or D8 resulted in the exclusive nuclear and cytoplasmic localization of ICP0, respectively (Figure 2), in agreement with previous studies [19] [45]. To confirm that ICP0 expressed from these mutants retained their predicted biological function, we measured the ability of each mutant virus to degrade promeylocytic leukemia protein (PML), as PML degradation is a well-characterized feature of nuclear ICP0. As expected, infection with R7914 led to the degradation of PML [19] while infection with D8 did not [45] (Figure 3). With respect to IRF3 activation, infection with D8 and R7914 mirrored that of WT and ICP0-null virus, respectively, in that IRF3 phosphorylation, dimerization and nuclear translocation were inhibited by cytoplasmic ICP0 only (Figure 4A and B). Although the kinetics of IRF3 dimerization between the ICP0-null mutant and R7914 appear to differ slightly, this is not a consistent observation (data not shown). Similar effects on IRF3 localization were observed following infection of two additional human primary fibroblasts cells (MRC-5 and BJ; data not shown). Interestingly, the early activation of IRF3 observed with WT HSV-1 (Figure 1) was not seen following infection with D8, likely owing to the early and exclusive cytoplasmic localization of ICP0 from this mutant. The rescued version of R7914, namely R7915 [46], demonstrated the same properties as the WT F strain HSV-1 in all respects (data not shown). In addition, experiments conducted with the D8 parental strain (17syn) demonstrated similar results to the F strain virus (data not shown). Although phosphorylation of IRF3 precedes dimerization and nuclear translocation [10], [47], [48], we were only able to efficiently detect phospho-IRF3 at 5 hpi. The sustained IRF3 activation observed following either ICP0-null or R7914 infections correlated with ISG56 production (Figure 4C). We consistently observed weak detection of ICP0 on western blots following infection with the R7914 mutant but intense levels following D8 infection (Figure 4C). This difference in ICP0 detection was determined to result from the protein extraction protocol employed in our studies, which favored the isolation of cytoplasmic proteins (Figure S1). In summary, these data support the hypothesis that cytoplasmic and not nuclear ICP0 is responsible for inhibiting IRF3 activation during HSV-1 infection.

**Figure 5:**
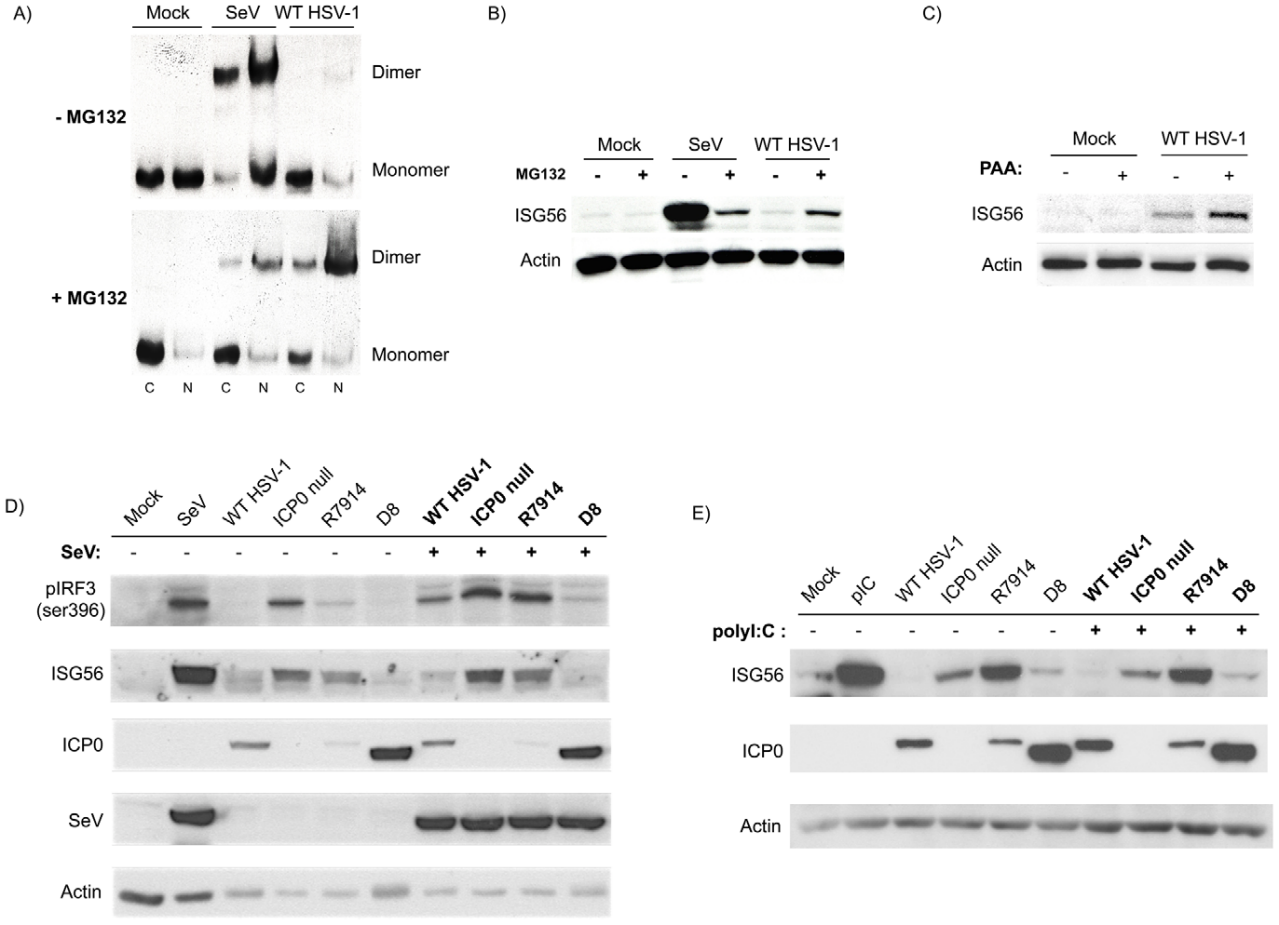
Nuclear retention of ICP0 blocks inhibition of IRF3 activation and ISG induction during WT infection. (A) HEL cells were mock treated or infected with WT HSV-1 for 8 hours in the absence or presence of MG132. IRF3 dimerization was examined by native western blotting in cytoplasmic and nuclear protein extracts. (B) Whole cell lysates were collected following an 8 hour infection as indicated. ISG56 levels were measured by western blot. In parts A & B, SeV was employed as a positive control for the activation of IRF3. (C) ISG56 expression was examined in protein lysates collected from HEL fibroblasts following an 8 hour infection with WT HSV-1 in the absence or presence of PAA. (D and E) Cytoplasmic ICP0 is capable of inhibiting activation of the IRF3 pathway following treatment with SeV or polyI:C. HEL cells were infected with WT HSV-1 or an ICP0 mutant virus for 12 hours and then treated with SeV (D) or polyI:C (E) for 8 hours. Expression of the indicated target proteins was examined by western blot.

Proteasome inhibitors, such as MG132 and lactacystin, as well as the viral DNA replication inhibitor phosphonoacetic acid (PAA), mediate ICP0 nuclear localization and retention [19], [25], [49]. These reagents were used to confirm the importance of ICP0 localization with respect to IRF3 inhibition. Nuclear retention of WT ICP0 by MG132 as observed in Figure 3, panel II, prevented ICP0 from blocking IRF3 activation and subsequent ISG56 induction (Figure 5A and B). Similar results were observed with lactacystin (data not shown). Nuclear retention of ICP0 by PAA also blocked the inhibition of IRF3-mediated ISG induction (Figure 5C). In summary, these results further support our hypothesis that cytoplasmic but not nuclear ICP0 inhibits IRF3 activation. Since PAA retains ICP0 within the nucleus in a proteasome-independent manner [50], [51], [52], [53], this observation suggests that in the presence of WT ICP0 and a functional proteasome, nuclear retention of ICP0 restricts its ability to block IRF3 activation during the early stages of an HSV-1 infection.

Studies utilizing a co-infection model or an inducible ICP0 expression model provide contradictory evidence regarding the ability of ICP0 to block IRF3 activation by an exogenous source [35], [37]. As a result, we examine the requirement of ICP0 localization during the inhibition of SeV- or polyI:C-mediated IRF3 activation using the ICP0 mutant viruses, R7914 and D8. Fibroblasts were pre-infected with either WT or ICP0 mutant HSV-1, and then subsequently treated with SeV or polyI:C for 8 hours before IRF3 activation was examined. Despite a higher overall level of IRF3 activation following SeV infection, WT and D8 infection blocked ISG56 production whereas ICP0-null and R7914 infection did not (Figure 5D). Similar results were observed when cells were treated with polyI:C (Figure 5E), with the exception that phospho-IRF3 was not readily observed following polyI:C treatment, consistent with published data [39]. Collectively, these results demonstrate a requirement for ICP0 cytoplasmic localization during the inhibition of IRF3 by a variety of exogenous stimuli.

### Proteasome activity modulates ICP0 localization but is not required for ICP0-mediated inhibition of IRF3

**Figure 6:**
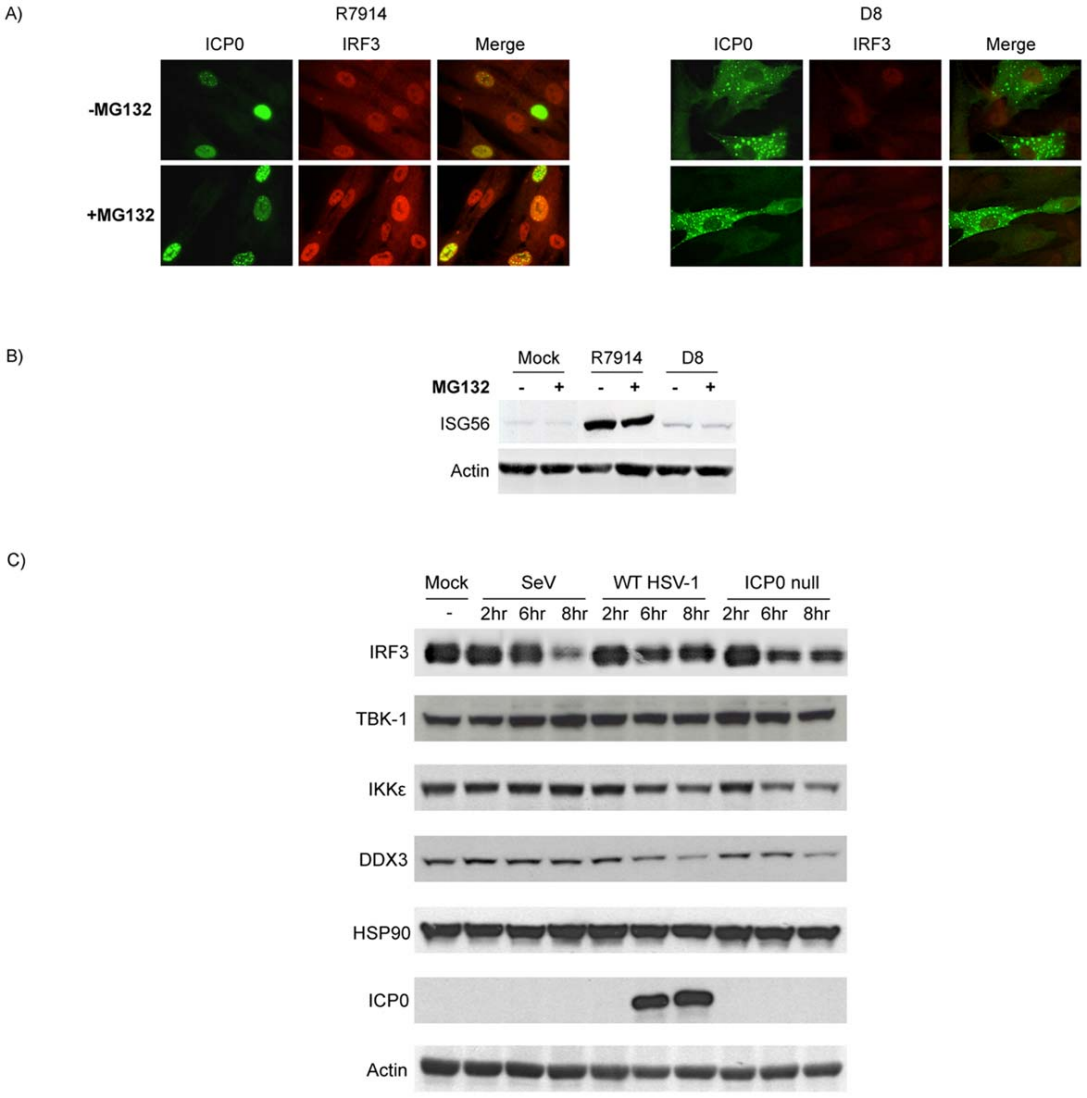
Proteasome activity facilitates appropriate subcellular localization of ICP0 and not degradation of IRF3 constituents. (A) Immunofluorescence microscopy was used to examine the localization of ICP0 and IRF3 in HEL cells following an 8 hour infection with R7914 or D8 in the absence or presence of MG132. (B) HEL cells were treated as described in part A and ISG56 expression was assessed by western blot analysis. (C) The expression of IRF3 pathway constituents was examined by western blot analysis following a time course infection of HEL cells with WT HSV-1 (strain F) or an ICP0 null virus (R7910). SeV was employed as a positive control for the activation of IRF3.

Since inhibition of cellular proteasome activity precludes ICP0 E3 ubiquitin ligase activity and restricts the localization of ICP0 to the nucleus [19], [49], we tested the effect of MG132 treatment on the activity of cytoplasmic ICP0. Following infection of fibroblasts with D8, MG132 did not alter the cytoplasmic localization of ICP0 or the ability of cytoplasmic ICP0 to block IRF3 nuclear accumulation (Figure 6A) or ISG56 induction (Figure 6B). The weak staining of IRF3 following D8 infection is consistent with studies suggesting that antibody detection of IRF3 favors its activated forms [35], [36], [54], [55]. As expected, no difference in ICP0 or IRF3 localization or ISG induction was observed following infection with R7914 in the presence or absence of MG132. We verified that the concentration of MG132 used effectively blocked proteasome activity by monitoring ICP0-mediated PML degradation [23], [25], [49] (Figure 3, panel II).

To further confirm that proteasome activity does not play a direct role in inhibiting IRF3 activation by ICP0, we examined the expression of IRF3 pathway constituents following WT and ICP0-null HSV-1 infection. As shown in Figure 6C, expression of IRF3, TBK-1, IKKε, DDX3 or HSP90 remain unchanged by the presence of ICP0 during infection. These data support and extend our original findings [33]. Collectively, these results suggest that the E3 ubiquitin ligase activity of ICP0 is not directly required for inhibiting IRF3-mediated ISG induction but rather functional proteasome activity is required to ensure proper ICP0 cellular localization.

## Discussion

Our current study highlights the importance of ICP0 subcellular localization and delineates the necessity of the proteasome during the inhibition of IRF3-mediated ISG induction. ICP0 is classically thought to perform the majority of its functions early in infection while localized in the nucleus [15], [16], [17]. To the best of our knowledge, a biological role for ICP0 in the cytoplasm has only been predicted based on the interaction and modification of elongation factor 1 δ [21]. In our current study, we found that cytoplasmic, but not nuclear, ICP0 inhibits IRF3-mediated ISG induction. This conclusion is based on the cumulative findings from diverse experimental systems, which included the use of genetic mutants or chemical inhibitors that limit the subcellular localization of ICP0. Utilizing multiple approaches helped minimize the experimental caveats associated with viral mutants and chemical inhibitors. Collectively, these experiments provide evidence that in addition to the nuclear functions of ICP0, cytoplasmic roles for ICP0 also exist.

Several studies have established that the entry of HSV-1 into host cells elicits activation of an IRF3-dependent antiviral response [4], [5], [6]. This response was demonstrated using UV-treated virus particles [3], [5], [56], [57], cycloheximide [4], [6], glycoprotein-deficient mutants [4], [5] or replication-deficient amplicon vectors [55]. In our current study, however, we observed activation of endogenous IRF3 during the early stages of infection with WT HSV-1 in human fibroblasts. These results support and extend a previous report by Preston and colleagues where they observed an IRF3-dependent response in selective fibroblast cell lines following WT HSV-1 infection [4]. Collectively, our studies support the idea that non-immune cells such as fibroblasts are capable of mounting innate antiviral responses against WT HSV-1 during the early stages of infection. We believe that this response is biologically relevant, particularly under conditions of low level virus exposure [57].

In agreement with the early activation of IRF3, we sometimes detect small amounts of ISGs following a WT HSV-1 infection (e.g. Figure 4C), a finding that others have reported as well [4]. This level of ISG induction, however, is insufficient to combat a high multiplicity infection. Accordingly, we propose that the cellular response to HSV-1 elicits a small amount of ISGs due to the early activation of IRF3 but accumulating cytoplasmic ICP0 prevents the sustained activation of IRF3, thus limiting the production of ISGs and offering a survival advantage to the virus. However, under conditions where the MOI of HSV-1 is low, the production of ISGs that ensues may outweigh the inhibitory effects of a sub-threshold amount of ICP0, thereby giving the host a survival advantage. The interaction between virus and host is a dynamic process, and a successful outcome will be determined by the species that is able to endure the battle between the production of cellular antiviral factors and viral immune evasion proteins.

It is intriguing that ICP0 delays inhibition of the IRF3 pathway until it is localized to the cytoplasm. Of interest, Everett and colleagues observed that depletion of PML, but not IRF3, increased plaque formation with an ICP0-deleted HSV-1 [58]. Their data suggests that PML is predominantly responsible for repressing HSV-1 infection at the earliest stages of infection. During the course of infection, ICP0 circumvents PML-mediated repression while localized within the nucleus [49], [59], [60]. Data presented with R7914 has shown that nuclear-restricted ICP0 is capable of degrading PML [19] but under the same conditions we failed to observe an inhibition of IRF3 activation, suggesting that the inability to localize to the cytoplasm precludes the inhibition of IRF3 but not PML. Taken together, both PML and IRF3 likely play a role in limiting HSV-1 infection but they appear to act at different times during the course of infection, and in turn, HSV-1 counters these respective responses accordingly. Consistent with this hypothesis, we found that D8, which retains IRF3 inhibitory activity but not PML inhibitory activity, and R7914, which retains PML inhibitory activity but not IRF3 inhibitory activity, displayed similar growth kinetics, which were reduced relative to parental HSV-1, in primary human fibroblasts (data not shown). However, to truly appreciate the role of host innate immune factors in virus pathogenesis, the use of relevant model systems are required. While gene deleted cell lines are useful tools for *in vitro* analysis, they often do not recapitulate cellular events within a host. For example, although IRF3-null mice were significantly more vulnerable to viral infection than WT mice, fibroblasts harvested from IRF3-null mice displayed normal antiviral responses [61]. Despite considerable effort, no murine alphaherpesviruses have been identified [62], precluding the use of mice as a relevant model system to study the role of ICP0 in herpesvirus pathogenesis.

Previous studies argued a necessity for the proteasome during ICP0-mediated inhibition of ISG induction [33], [34], and our results do not argue this point. However, previous studies did not investigate the role of the proteasome in dictating ICP0 localization versus mediating E3 ubiquitin ligase activity. We failed to observe degradation of any known components of the IRF3 pathway following WT infection, either in this study or a previous report [33], suggesting that proteasome function may be indirect. Data from the present study support the hypothesis that the E3 ubiquitin ligase activity of ICP0 is required for the appropriate subcellular localization of ICP0 during the inhibition of IRF3 activation. Consistent with this observation, we previously demonstrated that the ICP0 RING finger mutant FXE was unable to inhibit IRF3-mediated ISG induction [33]. While we did not monitor the localization of ICP0 within our previous study, others have shown that ICP0 RING finger mutants localize predominantly within the nucleus [22], [63]. Therefore, inappropriate cellular localization of ICP0 RING finger mutants likely explains this observation. In our current study, we confirmed that the RING finger mutant FXE was unable to inhibit IRF3 activation and predominantly localized to the nucleus, consistent with previous reports (data not shown). Collectively, these data suggest that ICP0 possesses biological activities that are independent of its RING finger domain. Accordingly, studies performed with proteasome inhibitors in the context of an HSV-1 infection should consider both ICP0 localization and E3 ubiquitin ligase activity.

Although previous reports investigating the ability of ICP0 to block IRF3 activation and subsequent ISG induction appear controversial [35], [36], [37], ICP0 localization varied within these different model systems. Our data agrees with two of the studies, in that cytoplasmic, but not nuclear, ICP0 blocks IRF3 activation. Our observations differ, however, when considering the mechanism of ICP0 activity. Whereas in a SeV co-infection model, ICP0 appeared to elicit IRF3 degradation, we failed to observe degradation of any known components of the IRF3 pathway in the context of an HSV-1 infection. Furthermore, proteasome activity was not required for cytoplasmic ICP0 activity. As SeV induces the proteasome-mediated degradation of IRF3 at late times post-infection [10], [11], it is likely that these differences stem from the model systems used. Recent data has brought to light differences in IRF3 activation following recognition of different pathogenic stimuli [38], [39], [64]. Despite the differences in our conclusions, our data agree with the idea that ICP0 is capable of inhibiting IRF3 activation by exogenous stimuli such as SeV or polyI:C. These data suggest that the component of the IRF3 pathway inhibited by ICP0 is common to the signaling cascades activated by each of the stimuli tested (HSV-1, SeV, or polyI:C), and most likely occurs upstream of IRF3 phosphorylation.

At this time, the exact mechanism utilized by ICP0 during the inhibition of IRF3 activation remains unclear. Given its role as a transcriptional activator, one possible mechanism may include that ICP0 facilitates the expression of a late viral gene product that in turn elicits IRF3 inhibitory effects. However, this possibility seems unlikely given that ICP0 is not essential for viral gene expression at the MOIs employed in our study [65], yet ICP0-null mutants failed to inhibit IRF3 activation. Accordingly, we failed to identify significant differences in early (ICP8) or late (gB) viral gene expression between WT and ICP0 null HSV-1 at the MOIs used in our study (Data not shown). In agreement with other studies [33], [34], ICP0 is necessary and sufficient to inhibit IRF3 activation during WT HSV-1 infection. We attempted to verify our results using ICP0 expression plasmids but were limited in that over-expression of WT or mutant ICP0 localized within the nucleus of fibroblasts (including the D8 mutant) and had strong toxic effects in this non-immortalized cell line (Figure S2). Similar observations of nuclear retention of exogenously delivered ICP0 have been reported elsewhere [37], [45], and likely reflect the growing evidence that additional viral proteins are required for the transition of ICP0 from the nucleus to the cytoplasm during viral infection [19].

We are currently investigating the mechanism employed by cytoplasmic ICP0 to inhibit IRF3 activation. Given that ICP0 does not lead to degradation of IRF3 pathway constituents, we speculate that ICP0 may inhibit IRF3 activation in a manner similar to USP7-mediated inhibition of TLR responses [41], wherein ICP0 recruits a protein that in turn elicits the inhibitory effect. Experiments are being conducted to identify cytoplasmic partners of ICP0 that may carry out such a role. Another avenue of investigation centers on the knowledge that ICP0 is highly modified as it has been shown to homodimerize [66], [67] as well as undergo phosphorylation and nucleotidylylation [29], [68], [69]. Posttranslational modifications are known to play a role in the appropriate subcellular localization of ICP0 as well as affecting transactivating and E3 ubiquitin ligase activity [30], [31]. The modifications of ICP0 required during the inhibition of IRF3-mediated ISG induction are of interest, especially in light of data showing that ICP0 is differentially modified in the nucleus as compared to the cytoplasm [29]. Therefore, in addition to subcellular localization, the post-translational modifications that ICP0 undergoes in each of the respective subcellular compartments likely also play a role in dictating function. An additional observation from these studies is the disappearance of activated forms of IRF3 concomitant with ICP0 cytoplasmic localization. It is unclear at this time whether ICP0 directly affects the loss of activated forms of IRF3 or whether activated IRF3 is relatively short-lived and the loss of nuclear IRF3 reflects the lack of newly activated molecules translocating into the nucleus.

Altogether, this study adds to our understanding of HSV-1 virus:host interactions and the role of ICP0 in inhibiting innate antiviral responses mediated by IRF3. Furthermore, this study stresses the importance of localization as a factor that influences the function of ICP0 but also highlights the importance of identifying post-translational modifications or additional activities that facilitate the functions of ICP0.

## Materials and Methods

### Reagents

MG132 (Sigma) was used at a concentration of 5 µM. An equal volume of DMSO (Sigma) was used as a vehicle control. MG132 was added to cultures 30 minutes prior to infection and remained within the cultures for all steps. Polyinosinic/polycytidylic acid (polyI:C) (GE Healthcare), was added to media at a concentration of 100 µg/ml. Phosphonoacetic acid (PAA), an inhibitor of viral DNA polymerase [51], [52], [53], was added during infection and in all subsequent steps at a concentration of 400 µg/ml.

### Cell lines

Human embryonic lung (HEL) fibroblasts and U2OS cells were purchased from the American Type Culture Collection (ATCC) and maintained in DMEM supplemented with 10% FBS, 2 mM L-glu, 100 U/ml penicillin, and 100 µg/ml streptomycin (pen/strep). Vero cells (ATCC) were maintained in DMEM supplemented with 5% FBS with L-glu and pen/strep.

### Viruses and infections

The WT HSV-1 strains used in this study include KOS, F, and strain 17 syn+ (17syn). HSV-1 ICP0 mutant viruses are described in Table 1. All WT strains were grown on Vero cells while mutant viruses were propagated on U2OS cells with 3 mM HMBA. All viruses were purified over a 36% sucrose cushion. All WT and mutant HSV-1 infections were performed with a MOI of 10 PFU/cell, unless otherwise stated, for 1 hour in serum-free media at 37°C. Infections with SeV (Cantell strain; Charles River Laboratories) were performed at 80 HA units per 10^6^ cells.

**Table 1:** Description of ICP0 mutant viruses employed in this study.

Virus	Mutation	Phenotype	Reference
**kos strain**			
n212	Insertion of a linker containing a nonsense mutation at residue 212	ICP0 null virus	[72]
**f strain**			
R7910	Complete deletion of both copies of the ICP0 gene	ICP0 null virus	[20]
R7914	Aspartic acid substituted for alanine at amino acid position 199	Nuclear retention of ICP0	[46]
**glasgow strain 17 syn**			
dl1403	2 Kb deletion in both copies of the ICP0 gene	ICP0 null virus	[73]
D8	Deletion of amino acids 475–548 (nuclear localization signal)	Cytoplasmic retention of ICP0	[63]

### Protein sample preparation

#### Whole cell extract protocol

Cells were washed twice and collected in PBS. Following centrifugation at 3000 rpm for 5 minutes, cell pellets were incubated in whole-cell extract buffer [3] for 15 minutes on ice followed by centrifugation for 10 minutes at 12,000 rpm. Protein quantification was performed using a Bradford assay kit (Bio-Rad Laboratories). Samples were stored in SDS-loading dye.

#### Cytoplasmic & nuclear protein extraction protocol

Cell extracts were washed with PBS followed by incubation in a hypotonic buffer [70] for 10 minutes on ice. Hypotonic buffer supplemented with 5% Triton X-100 was added to the cell extracts before centrifugation at 12,000 rpm for 3 minutes at 4°C. The supernatant (cytoplasmic protein) was mixed with loading dye (0.5 M Tris, pH 6.8; 20% glycerol; bromophenol blue). The pellet was incubated in a high salt buffer [70] for 30 minutes on ice and centrifuged at 12,000 rpm for 15 minutes at 4°C. The supernatant (nuclear protein) was mixed with loading dye. Protein quantification was performed using a Bradford assay kit (Bio-Rad Laboratories).

### Western Blotting

Whole cell protein extracts were resolved using denaturing polyacylamide gel electrophoresis and blocked in 5% skim milk. Blots were incubated with either rabbit anti-ISG56 (provided by G. Sen, Cleveland Clinic), rabbit anti-IRF3 (Santa Cruz Biotechnology), rabbit anti-phospho IRF3 (ser-396) (Cell Signalling), rabbit anti-TBK-1 (Millipore), rabbit anti-IKKε (Abcam), rabbit anti-DDX3 (Bethyl laboratories), rabbit anti-SeV [71], mouse anti-HSP90 (R&D Systems), mouse anti-ISG15 (provided by Dr. Borden, Cleveland Clinic), mouse anti-ICP0 (Virusys corporation), or goat anti-Actin (Santa Cruz Biotechnology). Membranes were subsequently probed with an HRP-conjugated goat anti-rabbit, goat anti-mouse, or rabbit anti-goat secondary antibody. Protein bands were visualized with ECL western blotting reagent (GE Healthcare Life Sciences).

### Native western blotting

Cytoplasmic and nuclear protein extracts were resolved on a non-denaturing gel in a buffer containing 25 mM Tris, pH 8.4 and 192 mM glycine with 0.2% deoxycholate present only in the cathode chamber. Membranes were blocked in 5% skim milk and subsequently incubated with rabbit anti-IRF3 (Immuno-Biological Laboratories, Co. Ltd.). Membranes were probed with HRP-conjugated goat anti-rabbit secondary antibody and protein bands were visualized with ECL western blotting reagent.

### Immunofluorescence Microscopy

Cells were grown to 50% confluency on coverslips. Following treatment, cells were fixed with 4% paraformaldehyde, permeabolized with 0.1% Triton X-100 and blocked with 2% goat serum. Cells were incubated with mouse anti-ICP0 and/or rabbit anti-IRF3 primary antibodies then incubated with anti-mouse or anti-rabbit Alexa fluor-conjugated secondary antibody (Invitrogen). Nuclei were stained with Hoechst dye (Dapi). All images were taken using a Leica DM-IRE2 microscope and analyzed using Openlab software (Improvision).

## Supporting Information

Figure S1 Proteins retained within the nucleus are difficult to detect by western blot analysis. (A) Immunofluorescence microscopy was used to examine the localization of ICP0 in HEL fibroblasts following an 8 hour infection with WT HSV-1 (F strain) in the absence or presence of PAA. (B) Western blot examining ICP0 expression following the collection of whole cell protein lysates from HEL fibroblasts following an 8 hour infection with WT HSV-1 in the absence or presence of PAA.
(2.71 MB TIF)

Figure S2 ICP0 expressed following plasmid transfection localizes to the nucleus. Immunofluorescence microscopy was used to examine the localization of IRF3 and ICP0 in HEL fibroblasts following transfection of expression plasmids encoding wild type (WT), RING finger mutant (FXE) or NLS mutant (D8) versions of ICP0.
(6.17 MB TIF)
