# Supplementary figures and images for: Cellular Localization of the Herpes Simplex Virus ICP0 Protein Dictates Its Ability to Block IRF3-Mediated Innate Immune Responses
Source: PLoS One. 2010 Apr 29;5(4):e10428. doi: 10.1371/journal.pone.0010428 (PMC2861674; doi:10.1371/journal.pone.0010428)

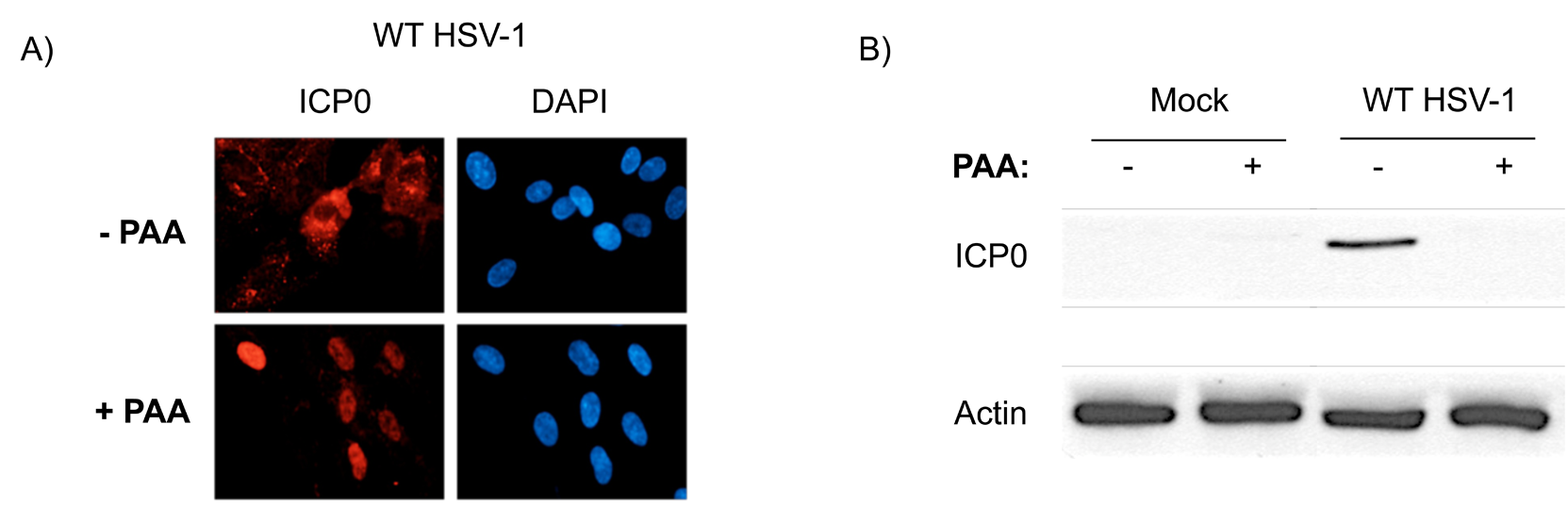

Supplement: Figure S1 — Proteins retained within the nucleus are difficult to detect by western blot analysis. (A) Immunofluorescence microscopy was used to examine the localization of ICP0 in HEL fibroblasts following an 8 hour infection with WT HSV-1 (F strain) in the absence or presence of PAA. (B) Western blot examining ICP0 expression following the collection of whole cell protein lysates from HEL fibroblasts following an 8 hour infection with WT HSV-1 in the absence or presence of PAA. (2.71 MB TIF) [file pone.0010428.s001.tif]

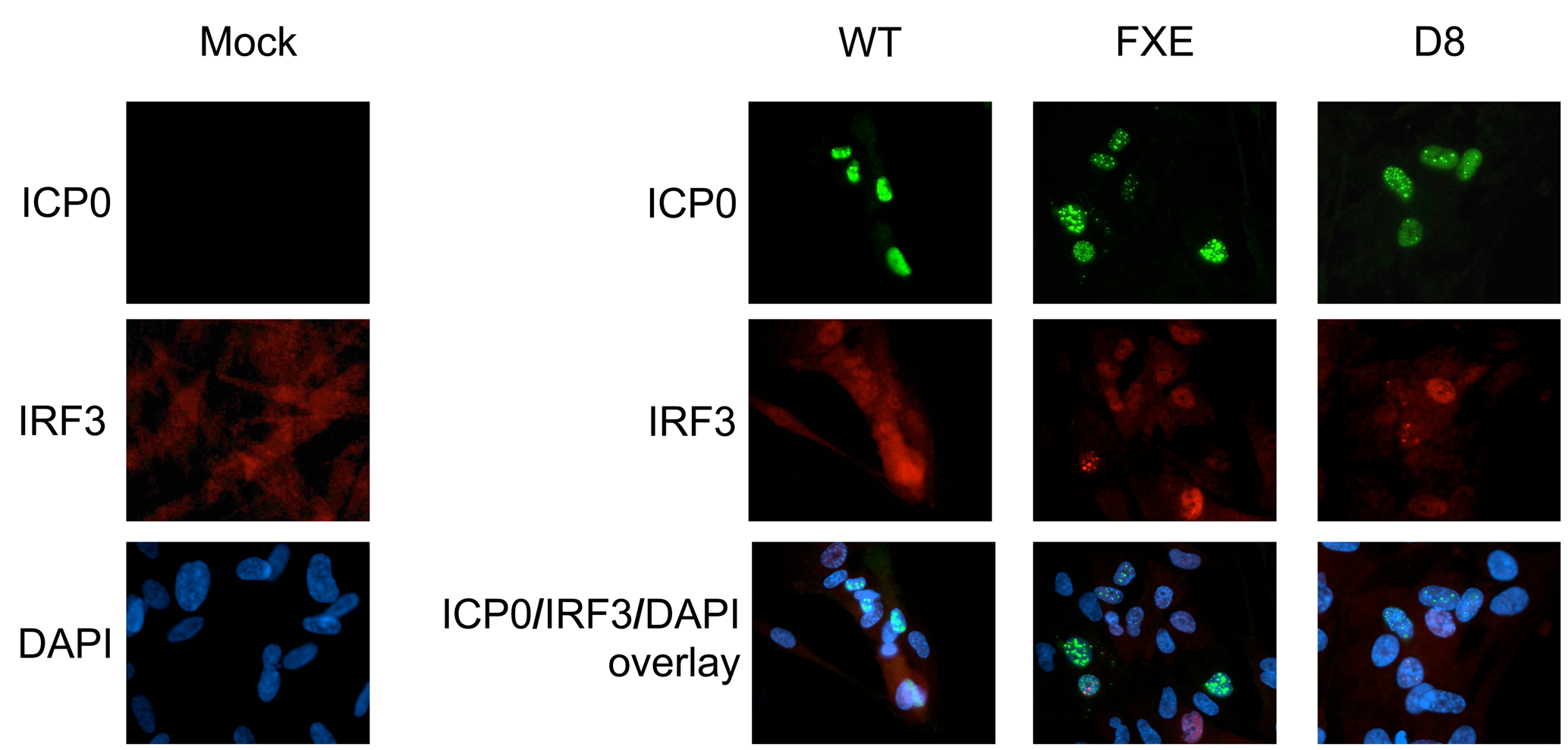

Supplement: Figure S2 — ICP0 expressed following plasmid transfection localizes to the nucleus. Immunofluorescence microscopy was used to examine the localization of IRF3 and ICP0 in HEL fibroblasts following transfection of expression plasmids encoding wild type (WT), RING finger mutant (FXE) or NLS mutant (D8) versions of ICP0. (6.17 MB TIF) [file pone.0010428.s002.tif]
